# Assessment and Management of Pain and Fever in Pediatric Patients in the Emergency Setting: A Proposed Flowchart for the Triage Nurse

**DOI:** 10.3390/children12101409

**Published:** 2025-10-18

**Authors:** Franca Benini, Stefano Masi, Luigi Martemucci, Patrizia Botarelli, Vincenzo Tipo, Tiziana Zangardi

**Affiliations:** 1 Paediatric Palliative Care, Pain Service Department of Women’s and Children’s Health, University of Padua, 35122 Padova, Italy; 2 Department of Health Sciences, University of Florence, 50121 Florence, Italy; 3 Department of Emergency Medicine, Anna Meyer Children’s University Hospital, 50139 Florence, Italy; 4 Department of General and Emergency Pediatrics, AORN Santobono-Pausilipon, 80122 Naples, Italy; 5 Pediatric Emergency Department, Santobono-Pausilipon Children’s Hospital, 80129 Napoli, Italy; 6 Pediatric Emergency Care Unit, Department of Woman’s and Child’s Health, University Hospital of Padua, 35128 Padua, Italy

**Keywords:** pain, fever, symptom assessment, disease management, pediatrics, emergency nursing, triage

## Abstract

**Background**: The nurse-initiated administration of medications, such as paracetamol and ibuprofen, at the time of triage may provide the opportunity to treat pediatric patients more quickly. We aimed to create practical flowcharts that can be used by all EDs with a specific focus on the nurse-initiated administration of medications to optimize the assessment and management of pain and fever in pediatric patients. **Methods**: Three regional expert meetings were held with a restricted working group composed of three chairmen and a wider working group composed of Directors of Pediatric EDs and Directors of Pediatric Departments, along with the main regional key experts for child healthcare management. Existing protocols were collected in the main centers belonging to the three regions and a unique recommendation was elaborated by the restricted group. This was then discussed and revised during discussion in a wider group. **Results**: Two protocols were developed for the triage nurse, one for pain and one for fever present. Both are initiated with assessment of the child’s pain or fever, followed by a caregiver interview to determine eligibility for the administration of an analgesic/antipyretic. In the case of pediatric pain, an analgesic is administered by the nurse only when the pain is rated as 4–6 and in the absence of specific contraindications. For pediatric fever, an antipyretic should only be administered if the child’s temperature is ≥37.5 °C and in the presence of at least 1 sign of discomfort. If an analgesic/antipyretic is administered, patients should be re-assessed after 1 h, and pediatrician evaluation requested as appropriate. **Conclusions**: The proposed flowcharts for pediatric pain and fever combine stratification by risk and severity and incorporate the possibility for the prompt administration of an antipyretic/analgesic when indicated.

## 1. Introduction

Fever and pain in children are frequent reasons for access to the Emergency Department (ED). Children with fever account for as many as 20% of visits to EDs, with a wide spectrum of underlying conditions ranging from mild illnesses to severe bacterial and viral infections [1]. At the same time, pain is a leading cause of pediatric ED visits. In a study from Canada, for example, symptoms of pain were present in 59% of all pediatric patient visits at triage [2]. Nevertheless, it is often assessed, undertreated, and not adequately managed [3]. A survey in 2019 by Benini et al. highlighted that pain assessment in Italian EDs is often inadequate: among 46 facilities studied, only 26 (56%) consistently measured pain during the initial assessment, while 17 (37%) did so occasionally, primarily in cases of obvious pain or specific conditions [4]. These shortcomings are likely due to the fact that pediatric pain is difficult to assess and manage considering the large number of causes [5]. However, the accurate management of pain is crucial for correct diagnosis and treatment [6]. In the attempt to overcome undertreatment of pain, some hospitals have implemented mandatory pain assessment for all pediatric patients presenting to the ED [7].

Fever may arise from a wide range of etiologies, which include bacterial and viral infections, malignancies, and other inflammatory conditions [8]. Moreover, a cause cannot be identified in about 20% of children [9]. Despite the availability of national and international guidelines for management of pediatric fever, inappropriate drugs and drug combinations are still used to lower the patient’s temperature, which is likely related to the concept of fever-phobia [10]. Interestingly, it has been reported that, in pediatric patients fever is treated more promptly than pain in the emergency setting (54 min vs. 83 min), which has been attributed to general attitudes of undertreatment of pain and unfounded concerns regarding fever in children [11].

Paracetamol and ibuprofen are analgesics indicated for the management of mild to moderate pain in pediatric patients due to their effectiveness and ease of administration. These medications exhibit comparable efficacy (a dose of 15 mg/kg of paracetamol is equivalent to a dose of 10 mg/kg of ibuprofen), while differing in their associated adverse effects [12]. Commonly reported side effects of ibuprofen include gastrointestinal issues, such as nausea and abdominal pain. However, it may also cause renal and hepatic adverse effects, depending on the dosage and other health factors [13]. On the other hand, paracetamol is characterized by a good tolerability profile regarding gastrointestinal, renal, and cardiovascular health. Cases of hepatotoxicity have however been reported due to various medication administration errors such as overdosing, doubling the prescribed dose, administering the medication too frequently, or using paracetamol continuously for a duration of up to 24 days [14]. However, when taken at therapeutic doses, paracetamol does not produce undesirable effects on the liver [15].

International and national guidelines agree that paracetamol and ibuprofen are recommended medications for managing fever in children, with paracetamol being suitable from birth and ibuprofen from three months of age [16,17]. All guidelines concur that the use of these two antipyretics should be considered in cases of fever accompanied by signs of discomfort. Intriguingly, growing evidence has raised concerns within the medical and scientific community regarding the potential for ibuprofen to mask the symptoms of infections and potentially worsen outcomes. This has been demonstrated in cases of community-acquired pneumonia and varicella, as well as in other respiratory tract infections [18]. For these reasons the European Medicines Agency’s Pharmacovigilance Risk Assessment Committee (PRAC) has advised updating the product information for medications containing ibuprofen and ketoprofen to indicate that these drugs may obscure the symptoms of infections [18].

The nurse-initiated administration of medications, such as paracetamol and ibuprofen, at the time of triage may provide the opportunity to treat pediatric patients more quickly [19]. Moreover, medications administrated earlier in the emergency setting may correlate with higher throughput and the possibility of more rapid discharge [19]. In Italy, triage is the starting moment in the ED where a nurse has the task of evaluating and assigning a priority code to the patient [20]. For some Italian hospitals, internal protocols for triage nurse are well-established, but there is a lack of shared guidance at national level that allows a unique approach to treat the child with fever or pain. In fact, the organization of Italian EDs is highly heterogeneous, leading to significant variations in the management approaches for children during triage. For example, in a survey of Italian EDs, routine pain assessment at triage and in the emergency room was performed by only 26% of EDs, around one-third did not report the use of algometric scales, and 47% had no local protocols in place for treatment of pain [21]. In some cases, there are dedicated pediatric EDs; however, very often, a pediatric patient may be referred to an adult or general ED due to the lack of a specific pediatric ED. Consequently, they are evaluated by a pediatrician or pediatric nurse only after leaving the emergency room.

Given the need to ensure a consistent approach to care and the relevance of pain and fever in pediatric patients in the emergency setting, we aimed to create practical flowcharts that can be used by all EDs (pediatric and general) with specific focus on nurse-initiated administration of medications. Moreover, since in Italy nurses have a lesser role in administration of medications compared to other countries like the US, we aimed to find the rationale to favor nurse-initiated care in order to streamline emergency treatment. The overall goal is to optimize and unify assessment and management of pain and fever in pediatric patients and to develop shared protocols that are applicable to all ED settings.

## 2. Materials and Methods

Three regional expert meetings were held in Veneto, Tuscany, and Campania Italian regions as representative of different realities of North, Center, and South of Italy. During each meeting, the currently used protocols for managing fever and pain in pediatric patients were discussed, with efforts made to update, unify, and make them applicable for all centers of the region. Moreover, the main unmet needs were also discussed and ideas on implementations for best practices were presented in a Round Table format. Emphasis was also placed on optimizing the patient’s journey and establishing a shared diagnostic and therapeutic approach. The outcomes from all three meeting were collated and discussed by a central steering committee, and the salient points were organized into algorithms for management of fever and pain in pediatric patients in an emergency setting, with focus placed on triage nurse.

A ‘focus group’ approach was used, during which 10 participants (all pediatricians) in Padua, 7 in Florence, and 10 in Naples gathered in their respective locations. The discussion of each group was guided and moderated by a chairman (a pediatrician). The insights that emerged from each group were combined into a single flowchart, reviewed and approved by each chairman. All participants were healthcare professionals who had taken part in local working groups on the management of pain and fever in the ED, along with presidents of scientific societies and heads of EDs and Pediatric Departments. The proposed protocols for the management of pediatric fever and pain at each meeting were analyzed, and best practices and unified flowcharts were developed to reflect the shared goals of the individual flowcharts. The starting point was from protocols/algorithms already in place in some of the centers participating in the round tables, along with sharing and implementing by the other participants which was then followed by a unanimous vote for acceptance.

The different phases of the project were:

• Definition of a restricted working group composed of three chairmen and a wider working group composed of Directors of Pediatric EDs and Directors of Pediatric Departments, along with the main regional key experts for child healthcare management;
• Literature search was performed using PubMed with keywords such as [pain], [fever], [child management], [pain score], [child emergency department], [paracetamol], [ibuprofen], [treatment of pain and fever], [assessment of pain and fever in children];
• Collection of existing protocols in the main centers belonging to the three regions;
• Elaboration of a unique recommendation by the restricted group;
• Discussion in the wider group;
• Revision of the recommendation according to what has emerged during the discussion phase;
• Comparison and alignment of the three recommendations to a unified indication available at a national level.

The current proposal required the approval of all members in the steering committee and was presented in plenary for approval.

## 3. Results

### 3.1. Protocol for Pain

**Figure 1:**
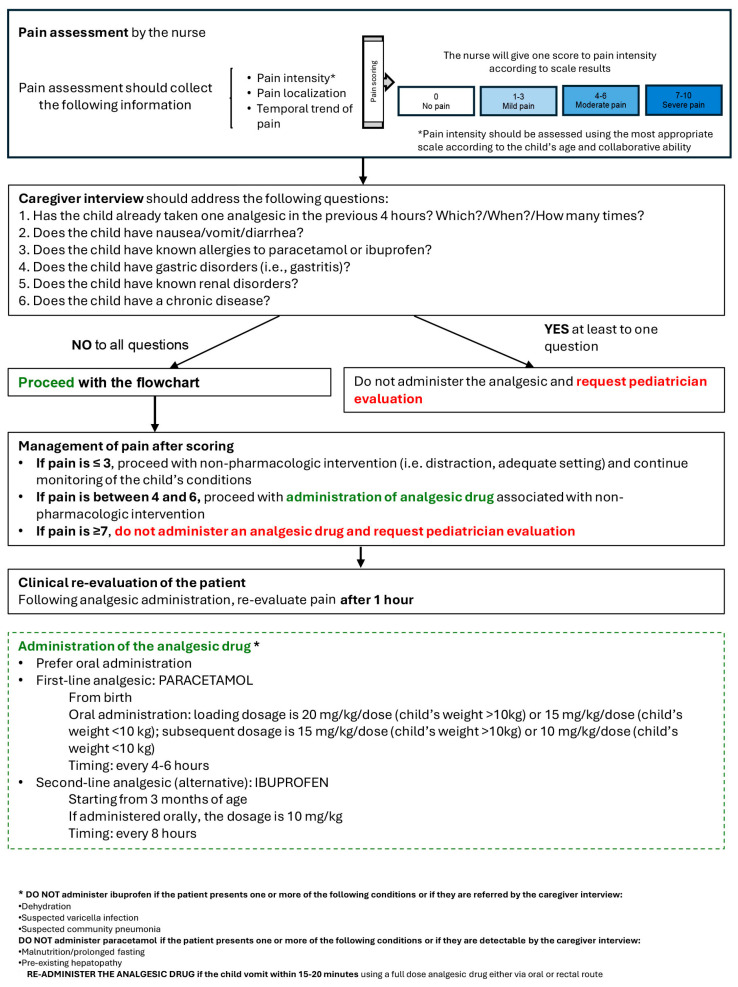
Pain flowchart. Proposed protocol for the triage nurse to assess and manage pain in pediatric patients.

The proposed protocol for the triage nurse regarding the assessment and management of pain in pediatric patients is shown in Figure 1. The protocol first consists of pain assessment, collecting information on the intensity, localization, and temporal trends in pain using the most appropriate scale according to the child’s age and characteristics. The suggested scales are detailed in Table 1 [22]. Suggestions for interventions are based on the guidelines of the Italian Pediatric Society [17] and the clinical practice of the participants. Recommended interventions are then based on the pain intensity score and caregiver and child (if possible, for age and clinical situation) interview. If the pain is ≤3, the nurse should proceed with non-pharmacologic intervention (i.e., distraction, adequate setting) and continue the monitoring of the child’s conditions. If pain is between 4 and 6, patient is eligible for administration of analgesic drug associated with non-pharmacologic intervention, while if the pain is ≥7 an analgesic should not be administered by the nurse and pediatrician evaluation should be requested. After that, the nurse should carry out a caregiver interview to collect information on the patient’s history and other presenting characteristics. If the child does not have any contraindication to an analgesic, as detailed in Figure 1, the nurse can administer an analgesic if indicated by the pain score. In administering analgesics, oral paracetamol should be preferred as first choice at a loading dose of 20 mg/kg/dose if child’s weight is >10 kg and 15 mg/kg/dose if ≤10 kg. Subsequent doses should be at 15 mg/kg/dose if the weight of the child is >10 kg and 10 mg/kg/dose if ≤10 kg, every 4–6 h. Ibuprofen can be considered as a second-line analgesic and should be administered at 10 mg/kg/dose every 8 h. Following administration of an analgesic, the pain should be re-assessed after 1 h, and if the pain did not decrease by at least 50%, consultation with a pediatrician is recommended. Of note, ibuprofen should not be administered if the patient is dehydrated, has suspected varicella infection or community pneumonia, or if these conditions are referred by the caregiver interview [23]. On the other hand, paracetamol should not be administered if the patient suffers from malnutrition/prolonged fasting, pre-existing hepatopathy or if these pathologies emerge from the caregiver interview, anamnesis, and available clinical data [24].

### 3.2. Protocol for Fever

**Figure 2:**
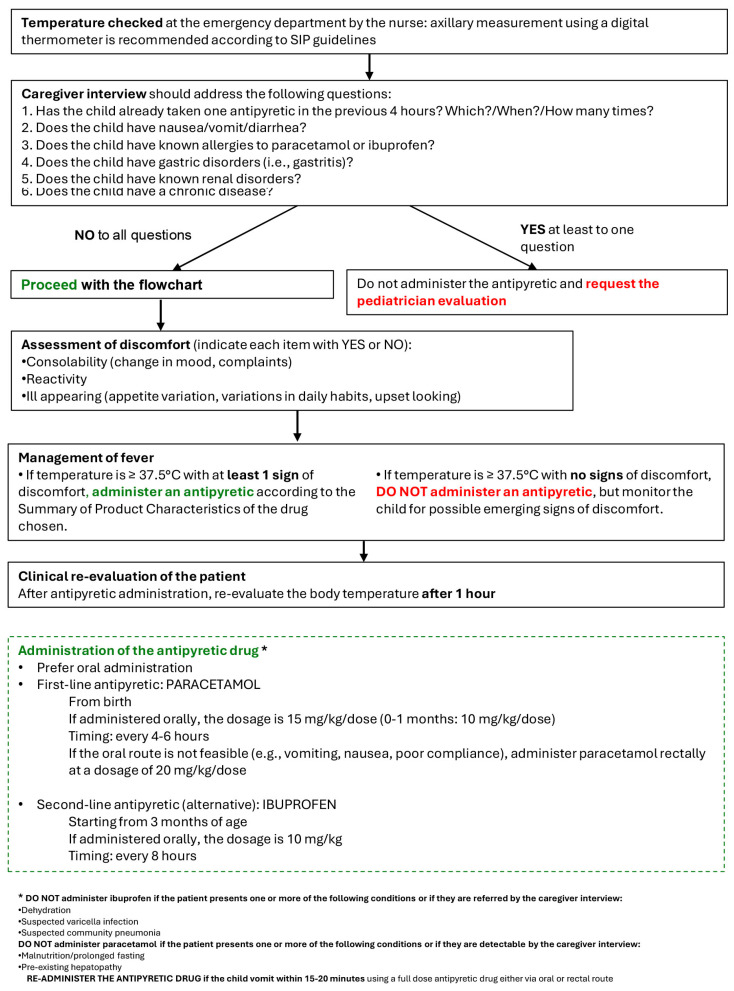
Fever flowchart. Proposed protocol for the triage nurse to assess and manage fever in pediatric patients.

The proposed protocol for fever management by a triage nurse is shown in Figure 2. The nurse begins by measuring the child’s temperature, preferably using an axillary method with a digital thermometer (in line with SIP guidelines). In accordance with the pain management protocol, the caregiver interview follows, which gathers information about the patient’s medical history and other relevant presenting characteristics. If an antipyretic is contraindicated by any of the items in the checklist, it should not be administered and evaluation by a pediatrician should be requested. If there are no contraindications to an antipyretic, the nurse proceeds with assessment of discomfort, considering three main signs: consolability (change in mood, complaints), reactivity, and ill appearance. If the child’s temperature is ≥37.5 °C with at least 1 sign of discomfort, the nurse should administer the antipyretic according to the Summary of Product Characteristics of the chosen drug. If the child’s temperature is ≥37.5 °C with no signs of discomfort, an antipyretic should not be given, although the child should continue to be monitored for emerging features of discomfort. When administered an antipyretic, paracetamol Via an oral route is preferred (15 mg/kg/dose every 4–6 h; 10 mg/kg/dose if 0–1 months old), while ibuprofen should be the second-line choice (10 mg/kg/dose every 8 h). Lastly, after administration of an antipyretic, body temperature should be re-assessed after 1 h. The contraindications for administration of either paracetamol or ibuprofen are the same as those for pain management.

## 4. Discussion

Given the high frequency of pediatric patients presenting with pain and fever in an emergency setting, three expert meetings were carried out with the goal of proposing practical flowcharts, applicable to all emergency settings, for nurse-initiated management of these two common symptoms. In this way, we aim to streamline patient assessment and provide rapid treatment when indicated during the triage phase.

Both protocols are initiated with assessment of the child’s pain or fever, followed by a caregiver and child (if possible, for age and clinical situation) interview to determine eligibility for administration of an analgesic/antipyretic. In the case of pediatric pain, an analgesic is administered by the nurse only when the pain is rated as 4–6 and in the absence of specific contraindications. For pediatric fever, an antipyretic should only be administered if the child’s temperature is ≥37.5 °C and in the presence of at least 1 sign of discomfort. If an analgesic/antipyretic is administered, patients should be re-assessed after 1 h, and pediatrician evaluation requested as appropriate. Standardized triage protocols for both pediatric pain and fever can help to reduce the heterogeneity of care currently offered in EDs across Italy and in other countries [21,25]. In this regard, the protocol for pain foresees the use of age-appropriate assessment tools. While the FLACC scale is suggested for children aged 0–3 years, for children with cognitive impairment it is recommended to use the FLACC Revised (r-FLACC) scale since it provides improved reliability and validity for assessment of pain in such children [26,27]. The r-FLACC has also been shown to be useful in young patients with spastic cerebral palsy [28].

Triage systems are vital tools for classification of patients presenting to an ED and help ensure that patients with more severe conditions are prioritized. This is needed since crowding and management of patient flow are some of the critical issues for EDs, leading to delays in treatment and adverse outcomes, as well as increased costs [29]. Pediatric emergency triage systems used globally have been divided into those involving risk stratification and rapid response [30]. Not all triage systems have been validated, and many have been adapted to the specific region in which their use is intended.

Nurse education is important in this regard, since nursing knowledge has been considered to be one of the barriers in assessing and treating pain in pediatric patients [31]. A pre/post intervention study reported that triage nurses in the ED are more likely to follow and implement standing orders for triage when they have received specific education on management of pain in pediatric patients, along with the importance of early intervention [31]. In addition, all nurses involved in triage of pediatric patients should undergo continuous education on children with fever in order to optimize outcomes and maintain the best standards of practice [32].

During the three expert meetings carried out in Veneto, Tuscany, and Campania regions involving the main experts of pediatric pain and fever in emergency settings, several key critical points emerged. First, the poor diffusion of uniform and shared advanced pediatric triage models among Italian hospitals became evident. Moreover, when in place, therapeutic algorithms and procedures might not be adhered to and monitoring of adverse events might not be carried out. In Italy at least, nurses traditionally have not had a role in choosing medications, which are usually only administered under orders from a physician, potentially creating a further barrier in patient management. As a consequence, pharmacological intervention may be delayed in the ED and in addition there is frequently the lack of dedicated personnel and adequately trained nurses. Assessment of discomfort is often not performed in a systematic manner despite the availability of established parameters and methods. Therefore, there is a need to clearly identify and unify which signs of discomfort should be reported and by which score they should be assessed. Additionally, the experts noted that the recommended drugs, paracetamol and ibuprofen, are often used at incorrect dosages often leading to apparent treatment failure or decreased pharmacological response. This highlights the importance of dose adjustment based on weight, although in the ED weight is not always measured, and paracetamol is often underdosed due to fear of intoxication. The flowcharts proposed take these key aspects into consideration and attempt to overcome these shortcomings.

## 5. Limitations

Among the limitations of the present proposal, it should be noted that the working group was small and unrepresentative, with only a few professionals in each focus group, and no nurses or healthcare directors; their involvement may be considered as part of a broader analysis in the future. This thus limits the representativeness and practical feasibility of the recommendations. The project also had no clear selection criteria which may have introduced bias, since those involved were already working in local groups and therefore potentially held similar views, reducing diversity of opinion. The focus group discussions were also described in general terms and information on how the data were recorded, coded, analyzed, and validated (e.g., the use of software, triangulation and double reading) is lacking, which is related to the small number of participants. In addition, only nurse-initiated administration of medications was taken into account, making it impossible to evaluate, the role of pharmacist-initiated administration or that by other healthcare professionals. Nonetheless, our focus was specifically on nurse-initiated triage in the emergency setting. It is also noted that proposed flowcharts have not yet been applied or tested in a real-world context, and have not been adapted to individual centers with a specific organization. Moreover, excessive overcrowding in emergency rooms combined with the lack of specialized personnel (the triage nurse) could prevent the application of the algorithms given the time needed to take the patient’s medical history and the time needed to train the triage nurse. There is also great heterogeneity of case studies regarding pain that does not always make it possible to apply a single model. In the future, the working group proposes to conduct follow-up assessments on the implementation and application of the flowcharts. This will include organizing education and training programs for triage personnel, along with seeking further approval from regional authorities for the protocols developed. Finally, the pediatricians involved wish to continue working on the project to integrate the flowchart into other stages of pediatric patient management, extending through to discharge from the ED.

## 6. Conclusions

The proposed flowcharts for pediatric pain and fever combine stratification by risk and severity and incorporate the possibility for the prompt administration of an antipyretic/analgesic when indicated.

## Figures and Tables

**Table 1:** Suggested scales for assessment of pain in pediatric patients [22].

Age/Characteristics	Suggested Pain Assessment Scales
0–3 years	Face, Legs, Activity, Cry, Consolability scale (FLACC scale *)
4–7 years	FLACC scale *, Faces Pain Scale—Revised (FPS-R)
≥8 years	Numerical Rating Scale, Faces Pain Scale—Revised (FPS-R)
Non-collaborative or with cognitive impairment *	Indirect assessment by caregiver and r-FLACC

* For children with cognitive impairment it is recommended to use the FLACC Revised (r-FLACC) scale. In children up to the age of 7 years, the FLACC is also recommended depending on the child’s understanding.

## Data Availability

No new data were created in this study.

## Abbreviations

The following abbreviations are used in this manuscript:

ED	Emergency Department
FLACC	Face, Legs, Activity, Cry, Consolability
r-FLACC	FLACC Revised
